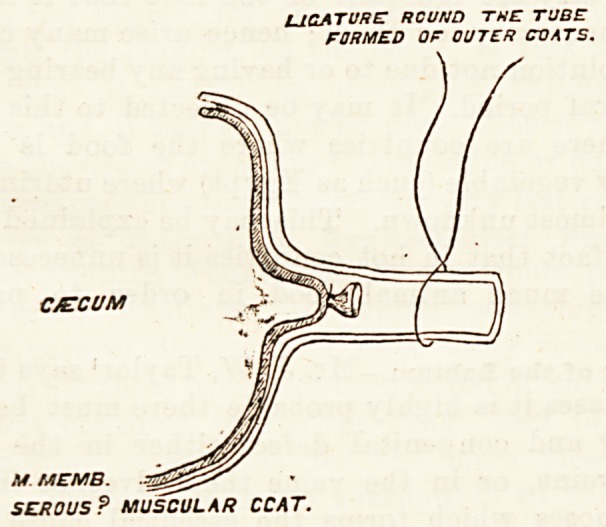# Progress in Surgery

**Published:** 1895-09-14

**Authors:** 


### Progress in Surgery.

### APPENDICITIS.

Murphy[1] holds that every case of appendicitis, promising or unpromising, should be treated by surgical operation at the earliest possible moment. This surgeon has operated on 194 cases, with a mortality of 9·6 per cent. When the abdominal wall is infiltrated and the abscess is opened without opening the unaffected portion of the peritoneum, there may be (*a*) a small circumscribed abscess, with the appendix forming part of the wall; (*b*) a small abscess, with a track leading to a deep-seated larger abscess; (*c*) a large abscess filling the iliac fossa, containing fæcal concretions, or a gangrenous appendix floating in the pus, closed in by firm adhesions; (*d*) there may be multiple abscesses with no connecting sinuses—a rare condition; (*e*) there may be an abscess in the opposite side of the abdomen. When the peritoneum is opened, and no adhesions to the anterior abdominal wall are found, a circumscribed abscess may exist in the posterior wall of the abdomen, the appendix and pus being enclosed within adherent omentum and intestine. This is the most common condition found in the early stage. Whenever the peritoneal cavity is opened directly, the appendix should be removed. Deaver[2] thinks that, as all inflammations of the appendix are septic, drainage is essential for recovery. Early operation is a conservative, and not a radical, procedure. The proportion of cases in which but one attack occurs and is followed by recovery is exceedingly small as compared with those in which the attack is repeated and a condition of invalidism exists in the interval. Stoker[3] considers in acute inflammation of the cæcum and its appendix: (1) That in this, as in some other acute peritoneal inflammations, operation is most unfavourable, should be seldom resorted to, and has been too freely adopted. (2) That purgation, if it can be induced, is the best remedy at our disposal; and that the most likely and safest way to effect it is by hydrostatic washing with warm water and a soft tube. White[4] thinks that if the symptoms are worse instead of better at the end of forty-eight hours, or earlier than that if there is severe sharp pain, increased tenderness and rigidity of the abdomen, and beginning tympany, either local or general, there can be no doubt that surgical interference offers by far the best hope of recovery. In the majority of cases these phenomena then indicate a perforation of the appendix wall, possibly not microscopic, but permitting the filtration through it of bacteria and their products. There is absolutely no way of recognising with any reasonable certainty which of these three events will follow: resolution and recovery; localised abscess, with from 90 to 95 per cent. of chances in the patient's favour; or general peritonitis with almost sure death if it is once well established. Although he believes more patients would be saved by operation at this time than by any temporising measures, experience has taught him to await events with more equanimity: (1) If the bowels are loose. (2) If the pain is dull and throbbing (connective tissue pain, Bryant), and not sharp and lancinating (serous tissue pain); the former he refers to a tense appendix with infiltration of the walls, but without gross perforation or intense or widespread peritonitis. (3) If the spot of greatest tenderness on pressure by a finger-tip is not precisely at McBurney's point. This is an empirical rule, but he has noticed in a number of cases in which there was delay at this stage, and which finally did well without operation, that the point of greatest tenderness was more or less remote from the usual region. (4) If vomiting is not marked. It will almost always be found present in an inverse relation to the looseness of the bowels. Its absence is a very favourable circumstance. (5) If, without marked change in the general condition, increased resistance, slight dulness, and the presence of a mass recognisable by palpation, indicate that a localised abscess is forming, shut off by adhesions from the geneal peritoneal cavity. As regards cases of relapsing appendicitis, White prefers operating in the quiescent period, and practically agrees with Treves that operation is indicated when (1) the attacks have been very numerous; (2) the attacks are increasing in frequency and severity; (3) the last attack has been so severe as to place the patient's life in considerable danger; (4) the constant relapses have reduced the patient to the condition of a chronic invalid, and have rendered him unfit to follow any occupation; and (5) owing to the persistence of certain local symptoms during the quiescent period there is a probability that a collection of pus exists in, or about, the appendix, Barker[5] advocates the following method of dealing with the appendix in cases where it is desirable to remove it: Its mesentery is first transfixed and tied in one or two parts with silk. The little mesentery is then cut with scissors up to the base of the appendix, close to the cæcum. Then, at about three-fourths of an inch from the latter, the serous and muscular coats are divided by a circular sweep of a sharp knife, leaving the mucous tube intact. The latter is now drawn out, and the two outer coats are stripped back towards the cæcum with a director, and turned over like the sleeve of a coat. In this way the tube of mucous membrane can be reached at its point of exit from the cæcum, and is tied with a fine silk ligature, and so closed. Then it is cut across an eighth of an inch beyond the ligature, and immediately retracts. The outer tube of serous and muscular tissue is now turned down over the stump of the mucous coats, simply surrounded with a fine silk or gut ligature, and closed over the mucous stump. Of course, the serous surfaces are not by this means brought together, but this is not necessary, as no firmer barrier to the prolapse of the mucous membrane could be made than by bringing the raw, inner, non-serous surfaces of the tube into contact. Stimson[6] discusses the behaviour of the appendix after a suppurative process has taken place, and the resulting abscess has been evacuated. The question arises from time to time whether it is advisable in such cases to make any search for the appendix, owing to the risk of breaking down adhesions which protect the abscess from the general peritoneal cavity; whether it is not best to leave the appendix and trust to its destruction by the process which has given rise to the abscess. In view of the fact that several cases are recorded where the appendix has subsequently caused serious mischief, in spite of one or two previous attacks of suppurative appendicitis, Stimson thinks we should not be in haste to be satisfied with the simple evacuation of an abscess which has formed about the appendix, but that search should be prosecuted with a view of removing the appendix, provided it is not attended by serious obstacles and dangers. Sahli[7] says every case of typhlitis with palpable swelling is attended by suppuration, but these cases, nevertheless, often recover, the pus partly being absorbed, and partly finding its way spontaneously into the interior of the bowel. When there is no improvement within three days an operation becomes necessary. An operation should be immediately performed only if the patient suffers from continuous fever, if he is seized with shivering, and if the temperature and pain suddenly increase after an apparently favourable initial stage of the disease. To avoid relapses the vermiform appendix must be removed, even if the pus is spontaneously evacuated into the intestines. Treves[8] records a further series of eighteen cases of relapsing typhlitis in which the operation has been performed during the quiescent period. Instances in which there is abiding tenderness and some swelling in the cæcal region, with very frequent attacks of pain and fever, are amenable to no measure short of operation. In all such cases he has found the appendix distended with pus. In examples not so marked such measures as the following may bring the attacks of typhlitis to an end: (1) The digestion must be attended to. (2) The bowels must be made to act daily. (3) Massage of the abdomen appears to have in many cases a very admirable effect, partly, it may be, by promoting the absorption of inflammatory exudations, and partly by encouraging a normal action of the bowels; with massage may be associated suitable exercise. (4) The use of some intestinal antiseptic. The most efficient would appear to be salol. It should be given in powder (in milk or in a cachet) in ten grain doses night and morning. Monod[9] and Ricard[10] both recommend operation as soon as the diagnosis of appendicitis is made. Roswell Park[11] has operated upon two cases of appendicitis, each caused by the presence of a pin in the appendix. McBurney[12] discusses the treatment of the diffuse form of septic peritonitis occurring as a result of appendicitis, and gives brief records of several cases upon which he has operated—several times successfully—for this condition. He thinks rubber tubes are inefficient, and that the best drainage in all parts of the body is the capillary drainage obtained by using absorbent dry material like gauze, and the more completely this material touches every point from which it is desirable to extract moisture, the more perfect will the drainage be. As regards washing out the pus cavity, he remarks that antiseptic fluids, and even hot sterile water, have a destructive effect upon the endothelium of the peritoneum. That is to say, the use of such fluids, while they serve to wash away septic material, tends to cripple the absorbing power of the peritoneum itself. No plan of treatment which could in the least degree deprive one of the valuable service of the peritoneum as an absorbing agent can be entirely satisfactory. He therefore uses normal salt solution at the body temperature. This fluid, used at this temperature and sterile, and of a uniform strength—six parts per thousand—has little or no destructive effect upon the peritoneal endothelium.

[1] Med. News, Jan. 5, 1895, and Brit. Med. Journ., Feb. 16, 1895. [2] Med. News, May 11, 1895, and Brit. Med. Journ., June 23, 1895. [3] Brit Med. Journ., June 1, 1895, p. 1,192. [4] Lancet, Feb. 16, 1895, p. 389. [5] Brit. Med. Journ., April 20, 1895. [6] Annals of Surgery, May, 1895. [7] Lancet, April 20, 1895. [8] Brit. Med. Journ., March 9, 1895, p. 517. [9] Therap. Gazette, Jan. 15, 1895. [10] Ibidem. [11] Med. Rec., New York, March 16, 1895. [12] Med. Rec., New York, March 30, 1895, p. 386.

## Figures and Tables

**Figure f1:**